# Outcomes Following Extracorporeal Membrane Oxygenation for Severe COVID-19 in Pregnancy or Post Partum

**DOI:** 10.1001/jamanetworkopen.2023.14678

**Published:** 2023-05-22

**Authors:** John J. Byrne, Amir A. Shamshirsaz, Alison G. Cahill, Mark A. Turrentine, Angela R. Seasely, Joe Eid, Caroline E. Rouse, Michael Richley, Nandini Raghuraman, Mariam Naqvi, Yasser Y. El-Sayed, Martina L. Badell, CeCe Cheng, James Liu, Emily H. Adhikari, Soha S. Patel, Erika R. O’Neil, Patrick S. Ramsey

**Affiliations:** 1 Department of Obstetrics and Gynecology, Division of Maternal-Fetal Medicine, University of Texas Health Science Center in San Antonio, San Antonio; 2 Department of Obstetrics and Gynecology, Baylor College of Medicine, Houston, Texas; 3 Department of Obstetrics and Gynecology, University of Texas at Austin, Dell Medical School, Austin; 4 Center for Women’s Reproductive Health, Department of Obstetrics and Gynecology, University of Alabama at Birmingham, Birmingham; 5 Department of Obstetrics and Gynecology, The Ohio State University College of Medicine, Columbus; 6 Department of Obstetrics and Gynecology, Division of Maternal-Fetal Medicine, Indiana University School of Medicine, Indianapolis; 7 Department of Obstetrics and Gynecology, University of California, Los Angeles; 8 Department of Obstetrics and Gynecology, Division of Maternal-Fetal Medicine, Washington University in St. Louis School of Medicine, St. Louis, Missouri; 9 Department of Obstetrics and Gynecology, Cedars Sinai Medical Center, Los Angeles, California; 10 Department of Obstetrics and Gynecology, Division of Maternal-Fetal Medicine and Obstetrics, Stanford University, Palo Alto, California; 11 Department of Obstetrics and Gynecology, Division of Maternal-Fetal Medicine, Emory University School of Medicine, Atlanta, Georgia; 12 Department of Obstetrics and Gynecology, Methodist Hospital, San Antonio, Texas; 13 Department of Obstetrics and Gynecology, Division of Maternal-Fetal Medicine, University of Cincinnati College of Medicine, Cincinnati, Ohio; 14 Department of Obstetrics and Gynecology, Division of Maternal-Fetal Medicine, University of Texas Southwestern Medical Center, Dallas, Texas; 15 Department of Obstetrics and Gynecology, Vanderbilt University Medical Center, Nashville, Tennessee; 16 Department of Pediatrics, Brooke Army Medical Center, San Antonio, Texas

## Abstract

**Importance:**

Existing reports of pregnant patients with COVID-19 disease who require extracorporeal membrane oxygenation (ECMO) are limited, with variable outcomes noted for the maternal-fetal dyad.

**Objective:**

To examine maternal and perinatal outcomes associated with ECMO used for COVID-19 with respiratory failure during pregnancy.

**Design, Setting, and Participants:**

This retrospective multicenter cohort study examined pregnant and postpartum patients who required ECMO for COVID-19 respiratory failure at 25 hospitals across the US. Eligible patients included individuals who received care at one of the study sites, were diagnosed with SARS-CoV-2 infection during pregnancy or up to 6 weeks post partum by positive nucleic acid or antigen test, and for whom ECMO was initiated for respiratory failure from March 1, 2020, to October 1, 2022.

**Exposures:**

ECMO in the setting of COVID-19 respiratory failure.

**Main outcome and measures:**

The primary outcome was maternal mortality. Secondary outcomes included serious maternal morbidity, obstetrical outcomes, and neonatal outcomes. Outcomes were compared by timing of infection during pregnancy or post partum, timing of ECMO initiation during pregnancy or post partum, and periods of circulation of SARS-CoV-2 variants.

**Results:**

From March 1, 2020, to October 1, 2022, 100 pregnant or postpartum individuals were started on ECMO (29 [29.0%] Hispanic, 25 [25.0%] non-Hispanic Black, 34 [34.0%] non-Hispanic White; mean [SD] age: 31.1 [5.5] years), including 47 (47.0%) during pregnancy, 21 (21.0%) within 24 hours post partum, and 32 (32.0%) between 24 hours and 6 weeks post partum; 79 (79.0%) had obesity, 61 (61.0%) had public or no insurance, and 67 (67.0%) did not have an immunocompromising condition. The median (IQR) ECMO run was 20 (9-49) days. There were 16 maternal deaths (16.0%; 95% CI, 8.2%-23.8%) in the study cohort, and 76 patients (76.0%; 95% CI, 58.9%-93.1%) had 1 or more serious maternal morbidity events. The largest serious maternal morbidity was venous thromboembolism and occurred in 39 patients (39.0%), which was similar across ECMO timing (40.4% pregnant [19 of 47] vs 38.1% [8 of 21] immediately postpartum vs 37.5% postpartum [12 of 32]; *P* > .99).

**Conclusions and Relevance:**

In this multicenter US cohort study of pregnant and postpartum patients who required ECMO for COVID-19–associated respiratory failure, most survived but experienced a high frequency of serious maternal morbidity.

## Key Points

**Question:**

What are outcomes associated with extracorporeal membrane oxygenation use during pregnancy or post partum in patients with COVID-19?

**Findings:**

In this multicenter retrospective cohort study that included 100 pregnant and postpartum patients, there were 16 maternal deaths, and most patients (76%) had 1 or more serious morbidity events.

**Meaning:**

Although most pregnant or postpartum patients who require extracorporeal membrane oxygenation for severe COVID-19 experience a serious morbidity event, these findings suggest that the vast majority will survive.

## Teaser

This cohort study investigates maternal and perinatal outcomes associated with extracorporeal membrane oxygenation (ECMO) used for pregnant patients with COVID-19 respiratory failure.

## Introduction

Prior research suggests pregnant patients with SARS-CoV-2 infection are at increased risk of severe disease at any time in the pregnancy compared with the nonpregnant state.^1,2^ Between 5% and 12% of pregnant patients who contract COVID-19 infection develop severe or critical disease.^3^ Patients who develop severe or critical COVID-19 are at increased risk for preterm birth, hypertensive disorders of pregnancy, venous thromboembolism, and critical care interventions such as mechanical ventilation and extracorporeal membrane oxygenation (ECMO).^4^

Historically, ECMO has been used in pregnant or postpartum patients with severe acute respiratory distress syndrome (ARDS) and refractory hypoxemia or cardiac failure as salvage therapy.^5,6^ During the 2009 H1N1 influenza pandemic, ECMO was used in pregnant patients who developed refractory hypoxemia resistant to conventional mechanical ventilation strategies.^7^ The role of ECMO in pregnancy for patients with ARDS secondary to H1N1 was examined in a systematic review and meta-analysis by Saad and colleagues,^7^ who reported a total of 39 patients with a maternal survival rate of approximately 75%. They concluded that due to a limited number of well-designed prospective studies, there is an uncertain benefit of using ECMO for maternal and neonatal survival. However, current evidence has advocated that the use of ECMO should be considered due to superior outcomes in pregnant or postpartum patients compared with nonpregnant patients.^8,9^

Reports of pregnant patients with COVID-19 who require ECMO are emerging. Several case reports and one large series of pregnant or peripartum patients with COVID-19 ARDS undergoing ECMO reported variable to no adverse outcomes for the maternal-fetal dyad.^8,10,11,12,13^ Some have noted 100% maternal survival,^8,10,12,13^ while other reports have noted up to 16% maternal mortality.^11^ This study aims to better understand the maternal and perinatal outcomes associated with ECMO used for COVID-19 respiratory failure in pregnancy and the postpartum period among a large, multicenter US cohort.

## Methods

We conducted a retrospective cohort study of pregnant or postpartum patients with singleton or multiple gestations placed on ECMO and delivered at 1 of the 25 hospitals. The 25 hospitals were affiliated with 1 of 22 centers spread geographically across the United States. Of the 25 hospitals involved in this study, 23 were associated with academic medical centers (University of Texas Health Science Center in San Antonio, San Antonio; Baylor College of Medicine, Houston, Texas; University of Texas at Austin, Dell Medical School, Austin; University of Alabama at Birmingham, Birmingham; The Ohio State University College of Medicine, Columbus; Indiana University School of Medicine, Indianapolis; University of California, Los Angeles, Los Angeles; Washington University in St. Louis School of Medicine, St. Louis, Missouri; Cedars Sinai Medical Center, Los Angeles, California; Stanford University, Palo Alto, California; Emory University School of Medicine, Atlanta, Georgia; University of Cincinnati College of Medicine, Cincinnati, Ohio; Parkland Health, Dallas, Texas; University of Texas Southwestern Medical Center, Dallas; Vanderbilt University Medical Center, Nashville, Tennessee; University of Kansas Medical Center, Kansas City; University of South Florida and Tampa General Hospital, Tampa; The University of Mississippi Medical Center, Jackson; Thomas Jefferson University Hospital, Philadelphia, Pennsylvania; University of North Carolina at Chapel Hill, Chapel Hill; University of California San Diego, La Jolla), 1 was a community hospital (Methodist Hospital, San Antonio, Texas), and 1 was a military hospital (Brooke Army Medical Center, San Antonio, Texas). The study sites involved were a convenience sample of study institutions across the US that were invited to participate. A steering committee oversaw the study, and this report follows the Strengthening the Reporting of Observational Studies in Epidemiology (STROBE) reporting guideline.^14^ The study was approved by each institutional review board at all institutions participating in the study, and data use agreements were obtained with the primary site at UT Health in San Antonio. A waiver of consent was granted by each institutional review board because it was a retrospective study.

All patients that were treated at 1 of the study sites were included in the study cohort if: (1) they received the diagnosis based on either a positive nucleic acid or antigen test result for SARS-CoV-2 virus during pregnancy or up to 6 weeks post partum, and (2) they were started on ECMO from March 1, 2020, to October 1, 2022. Due to limited testing early in the pandemic, patients who received ECMO for presumed SARS-CoV-2 infection were also included. Patients underwent venoarterial or venovenous ECMO during the study time frame.

Data on sociodemographic, obstetrical, perinatal, and maternal outcomes were extracted from the medical record. To best understand the population being studied, race and ethnicity were obtained from the electronic health record at each institution. The primary residence zip code was used to identify the Social Vulnerability Index, developed by the Centers for Disease Control and Prevention, to identify socially vulnerable populations using 15 US Census variables.^15^ The majority of patients were placed on anticoagulation for ECMO runs, however, full data on dosing and medication used was not abstracted. The primary study outcome was maternal mortality, defined as maternal death while on ECMO or within 30 days of discharge. Secondary outcomes included serious maternal morbidity events defined as any of the following: venous thromboembolism, ischemic stroke or intracranial hemorrhage, acute kidney injury, limb ischemia, gastrointestinal bleeding, liver failure, ischemic injury (noncardiac and nonintracranial), and cardiac complications (myocardial infarction, cardiomyopathy, arrhythmias, cardiogenic shock, cardiac arrest). Obstetrical secondary outcomes included obstetrical morbidity, specifically postpartum hemorrhage if delivered on ECMO, clinically diagnosed hypertensive disorders of pregnancy, placental abruption, preterm prelabor rupture of membranes, early pregnancy loss (miscarriage; pregnancy loss before 20 weeks of gestation), and stillbirth. Neonatal secondary outcomes included neonatal death, small for gestational age, birth weight less than 1500 g, birth weight less than 2500 g, 5-minute Apgar score 3 or less, neonatal intensive care unit admission, neonatal intubation, respiratory distress syndrome, transient tachypnea of the newborn, neonatal sepsis, interventricular hemorrhage, necrotizing enterocolitis, bowel perforation, bronchopulmonary dysplasia, and chronic lung disease.

Data were collected and managed using Research Electronic Data Capture (REDCap) software version 12.4.22 hosted at UT Health.^16,17^ The data was manually entered into REDCap by each site investigator. When data extraction was completed by the site, the primary investigator (J.J.B.) performed data checks of all data and resolved any discrepancies by querying site investigators. At the time of data extraction, information from 5 pregnant patients and 1 postpartum patient had been published in the form of 3 case series.^10,18,19^

Outcomes were compared according to timing of ECMO initiation relative to pregnancy or postpartum stage (pregnant, <24 hours post partum, ≥24 hours and within 6 weeks post partum), from this point forward referred to as pregnancy status, and periods of circulation of SARS-CoV-2 variants. The ECMO initiation stages were developed from prior literature on important time frames within the pregnancy and puerperium.^6^ The time epochs were based on SARS-CoV-2 dominant strain or variants in the US: March 2020 to December 2020 (wild-type), January 2021 to June 2021 (Alpha), July 2021 to November 2021 (Delta), and December 2021 to October 2022 (Omicron).^20^ Statistical tests included Fisher exact test, Kruskal-Wallis rank-sum test, and Mann-Whitney *U* test as appropriate. Two-sided *P* < .05 was considered statistically significant. Statistical analyses were performed using R version 3.6.3 (R Project for Statistical Computing).

## Results

Among the 100 patients included in this study who received ECMO for respiratory failure associated with SARS-CoV-2 infection during pregnancy or postpartum from March 1, 2020, through October 1, 2022, 29 (29.0%) were Hispanic, 25 (25.0%) were non-Hispanic Black, and 34 (34.0%) were non-Hispanic White; 47 were pregnant, 21 were within’ 24 hours post partum, and 32 were between 24 hours and 6 weeks post partum; and the mean (SD) age was 31.1 (5.5) years; most patients had obesity (79.0% [n = 79]), had public or no insurance (61.0% [n = 61]), and did not have an immunocompromising condition (67.0% [n = 67]) (Table 1). Of the 100 patients included, 14 were treated at centers with fewer than 4 cases reported, and 86 were treated at centers with 4 or more cases reported. A larger number of patients received ECMO during the Delta variant wave (48.0%) compared with the other variant waves (12.0% for wild-type, 27.0% for Alpha, and 13.0% for Omicron). Only 4 patients were previously vaccinated, 3 with BNT162b2 mRNA COVID-19 vaccine and 1 with mRNA-1273 SARS-CoV-2 vaccine. Of those vaccinated, 2 did not have any serious morbidity events, and 2 experienced venous thromboembolism, and 1 of those 2 had a cardiac arrhythmia. There were no maternal deaths among the 4 vaccinated patients.

**Table 1.** Demographic Characteristics of Individuals Requiring ECMO for COVID-19–Associated Respiratory Failure While Pregnant, Within 24 Hours Post Partum, and Within 6 Weeks Post Partum

Variable	Individuals, No. (%)			
	Total (n = 100)	Pregnant (n = 47)	Immediately post partum (n = 21)^a^	Post partum (n = 32)^b^
Age, mean (SD), y	31.1 (5.5)	31.2 (5.3)	30.7 (5.0)	31.3 (5.5)
BMI, mean (SD)	35.8 (8.4)	35.1 (8.2)	36.8 (7.7)	36.3 (9.5)
30 or higher	79 (79.0)	35 (74.5)	19 (90.5)	25 (78.1)
40 or higher	27 (27.0)	11 (23.4)	8 (38.1)	8 (25.0)
Race and ethnicity				
Hispanic	29 (29.0)	18 (38.3)	5 (23.8)	6 (18.6)
Non-Hispanic Black	25 (25.0)	7 (14.9)	3 (14.3)	15 (46.9)
Non-Hispanic White	34 (34.0)	15 (31.9)	12 (57.1)	7 (21.9)
Other^c^	12 (12.0)	7 (14.9)	1 (4.8)	4 (12.5)
Parity, median (IQR)	2 (1-3)	2 (1-3)	2 (1-3)	2 (1-3)
Obstetric history				
Previous preterm birth	12 (11.0)	6 (12.8)	3 (14.3)	1 (3.1)
Previous cesarean delivery	33 (33.0)	16 (34.0)	6 (28.6)	11 (34.4)
Previous hypertensive disorder of pregnancy	21 (21.0)	8 (17.0)	5 (23.8)	8 (25.0)
Public or no insurance	61 (61.0)	29 (61.7)	11 (52.4)	21 (65.6)
Smoked during this pregnancy	3 (3.0)	2 (4.3)	NA	1 (3.1)
Any substance use during this pregnancy	1 (1.0)	NA	1 (4.8)	NA
Blood type				
A	31 (31.0)	17 (36.2)	5 (23.8)	9 (28.1)
B	12 (12.0)	4 (8.5)	4 (19.0)	4 (12.5)
AB	4 (4.0)	2 (4.3)	1 (4.8)	1 (3.1)
O	53 (53.0)	24 (51.1)	11 (52.4)	18 (56.3)
Rh-positive	97 (97.0)	44 (93.6)	21 (100.0)	32 (100.0)
Immunocompromising condition				
Asthma or chronic obstructive pulmonary disease	12 (12.0)	6 (12.8)	3 (14.3)	3 (9.4)
Pregestational diabetes	11 (11.0)	8 (17.0)	1 (4.8)	2 (6.3)
Chronic hypertension	14 (14.0)	12 (25.5)	NA	2 (6.3)
Thyroid disease	4 (4.0)	2 (4.3)	NA	2 (6.3)
Vaccinated against SARS-CoV-2	4 (4.0)	2 (4.3)	1 (4.8)	1 (3.1)

Abbreviations: BMI, body mass index (calculated as weight in kilograms divided by height in meters squared); NA, not applicable.

^a^ Immediately post partum defined as less than 24 hours after delivery.

^b^ Post partum defined as at least 24 hours after delivery and within 6 weeks’ post partum.

^c^ Other race and ethnicity was defined as American Indian, Asian, Native Hawaiian or Pacific Islander, not disclosed, or more than 1 race.

The median (IQR) ECMO run was 20 (9-49) days. Of the 47 patients who were pregnant, the median (IQR) gestational age at the time of ECMO initiation was 25.1 (8.4-31.9) weeks. There were 16 maternal deaths (16.0%; 95% CI, 8.2%-23.8%) in the study cohort. Although there was a higher percentage of deaths in the postpartum cohort, the differences across the pregnancy status groups were similar (Table 2). Maternal death was no more likely to occur in patients hospitalized at centers with less than 4 ECMO cases during the study interval (5 deaths [35.7%] in centers with 3 or fewer cases vs 11 deaths [12.8%] in centers with 4 or more cases (*P* = .05) (eTable 1 in Supplement 1). In addition, maternal death occurred more frequently in individuals who experienced cardiac arrest (*P* < .001), acute kidney injury (*P* = .01), and intracranial hemorrhage (*P* = .03) (eTable 2 in Supplement 1). While 97 patients (97.0%) were intubated before ECMO initiation, 3 patients were not, and 5 ultimately required a lung transplantation.

**Table 2.** Serious Maternal Morbidity Events

Outcome	Individuals, No. (%)				*P* value^c^
	Total (n = 100)	Pregnant (n = 47)	Immediately post partum (n = 21)^a^	Post partum (n = 32)^b^	
Maternal death^d^	16 (16.0)	8 (17.0)	1 (4.8)	7 (21.9)	.20
Venous thromboembolism	39 (39.0)	19 (40.4)	8 (38.1)	12 (37.5)	>.99
Stroke	5 (5.0)	2 (4.3)	1 (4.8)	2 (6.2)	>.99
Acute kidney injury	27 (27.0)	11 (23.4)	4 (19.0)	12 (37.5)	.38
Limb ischemia	2 (2.0)	1 (2.1)	NA	1 (3.1)	>.99
Gastrointestinal bleeding	8 (8.0)	3 (6.4)	2 (9.5)	3 (9.4)	.80
Liver failure	1 (1.0)	NA	NA	1 (3.1)	.55
Ischemic injury (noncardiac or intracranial)	8 (8.0)	2 (4.3)	NA	6 (18.8)	.03
Cardiac complications^e^	38 (38.0)	16 (34.0)	6 (28.6)	16 (50.0)	.22

Abbreviation: NA, not applicable.

^a^ Immediately post partum defined as less than 24 hours after delivery.

^b^ Post partum defined as at least 24 hours after delivery and within 6 weeks post partum.

^c^ Fisher exact test.

^d^ Maternal death on ECMO or within 30 days of discharge.

^e^ Cardiac complications included myocardial infarction, cardiomyopathy, arrhythmia, cardiogenic shock, or cardiac arrest.

Among all 100 patients, 76 patients (76.0%; 95% CI, 58.9%-93.1%) had 1 or more serious maternal morbidity events. While hematologic, thromboembolic, or ischemic events were similar among the various pregnancy statuses, the highest serious morbidity was venous thromboembolic events. Venous thromboembolism occurred in 39 patients (39.0%), which was similar between the groups (40.4% pregnant [19 of 47] vs 38.1% immediately post partum [8 of 21] vs 37.5% post partum [12 of 32]; *P* > .99) (Table 2). Ischemic injuries were more likely to occur in the postpartum period (18.8% [6 of 32] post partum vs 4.3% [2 of 47] pregnant) (*P* = .03) (Table 2).

Rates of hypertensive disorders of pregnancy, placental abruption, preterm prelabor rupture of membranes, or perinatal death were similar across pregnancy status and variant waves. Early pregnancy loss, fetal or neonatal demise occurred in 13 patients (13.0%) who were pregnant or immediately postpartum at the time of ECMO initiation. Median (IQR) gestational age in weeks at delivery of all live births^21^ was 29.4 (28.2-31.6) weeks. Among the 23 patients (23.0%) who delivered while receiving ECMO, the median (IQR) gestational age was 29.0 (26.9-29.6) weeks, and 8 (34.8%) had a postpartum hemorrhage. No differences in neonatal outcomes were identified across pregnancy status except for a higher rate of bronchopulmonary dysplasia noted in babies whose mothers received ECMO in the immediate postpartum period and slightly higher birth weight in those born whose mothers received ECMO in the postpartum period (Table 3). Among the 59 live-born infants, the frequency of low birth weight (<2500 g) occurred in 47 (79.7%), neonatal ICU admission in 50 (86.2%), and neonatal intubation in 39 (68.6%) (Table 3).

**Table 3.** Obstetrical and/or Perinatal Outcomes

Variable	Individuals, No./total No. (%)				*P* value^c^
	Total (n = 100)	Pregnant (n = 47)	Immediately post partum (n = 21)^a^	Post partum (n = 32)^b^	
Hypertensive disorders of pregnancy	32/100 (32.0)	13/47 (27.7)	7/21 (33.3)	12/32 (35.3)	.76
Placental abruption	1/100 (1.0)	1/47 (2.1)	NA	NA	>.99
Preterm prelabor rupture of membranes	1/98 (1.0)	1/46 (2.2)	NA	NA	>.99
Early pregnancy loss	5/100 (5.0)	5/47 (10.6)	NA	NA	.08
Fetal demise	7/99 (7.1)	5/46 (10.9)	2 (9.5)	NA	.11
Neonatal demise	3/99 (3.0)	1/46 (2.2)	NA	2 (6.1)	.60
Live birth^d^	59/100 (59.0)	26/47 (55.3)	17/21 (81.0)	16/32 (50.0)	.06
Gestational age at delivery, median (IQR), wk	29.4 (28.2-32.1)	29.4 (27.7-31.6)	29.3 (28.3-30.1)	32.1 (28.7-37.1)	.06^e^
Small for gestational age	4/59 (6.8)	2/26 (7.7)	2/17 (11.8)	0	.56
Birth weight <1500 g	33/59 (55.9)	17/26 (65.4)	10/17 (58.8)	6/16 (37.5)	.23
Birth weight <2500 g	47/59 (79.7)	23/26 (88.5)	15/17 (88.2)	9/16 (56.3)	.04
Apgar <3 at 5 min	11/59 (18.6)	6/26 (23.1)	3/17 (17.6)	2/16 (12.5)	.84
Neonatal ICU admission	50/58 (86.2)	22/26 (84.6)	15/16 (93.8)	13/16 (81.3)	.71
Neonatal intubation	39/56 (69.6)	19/26 (73.1)	12/15 (80.0)	8/15 (53.3)	.32
Respiratory distress syndrome	39/56 (69.6)	19/26 (73.1)	11/15 (73.3)	9/15 (60.0)	.51
Transient tachypnea of the newborn	4/56 (7.1)	3/26 (11.5)	1/15 (6.7)	0	.47
Neonatal sepsis	3/56 (5.4)	1/26 (3.8)	1/15 (6.7)	1/15 (6.7)	>.99
Necrotizing enterocolitis	3/56 (5.4)	0	1/15 (6.7)	2/15 (13.3)	.09
Bowel perforation	1/56 (1.8)	0	0	1/15 (6.7)	.54
Bronchopulmonary dysplasia	10/56 (17.9)	4/26 (15.4)	6/15 (40)	0	.01
Chronic lung disease	6/56 (10.7)	4/26 (15.4)	1/15 (6.7)	1/15 (6.7)	.64

Abbreviations: ICU, intensive care unit; NA, not applicable.

^a^ Immediately post partum defined as within 24 hours after delivery.

^b^ Post partum defined as at least 24 hours after delivery and within 6 weeks post partum.

^c^ Fisher exact test.

^d^ As defined by US Centers for Disease Control and Prevention.^21^

^e^ Kruskal-Wallis rank-sum test.

## Discussion

In a large US cohort of pregnant and postpartum patients initiated on ECMO for COVID-19–associated respiratory failure, 16% of the patients died, and the risk of death was higher in patients who experienced a cardiac arrest, acute kidney injury, or intracranial hemorrhage. Pregnancy loss (early pregnancy loss or stillbirth) or neonatal mortality occurred in 13% of patients who were pregnant or immediately post partum at the time of ECMO initiation, and among survivors the majority had a birth weight of less than 2500 g, were admitted to the neonatal intensive care unit, and required intubation.

The results of this cohort study expand on the previously reported data on ECMO use in pregnancy for ARDS. In a systematic review and meta-analysis reviewing ECMO use for ARDS secondary to H1N1 infection in pregnant and postpartum patients, the pooled survival rate was as high as 74.6%.^13^ When compared with all etiologies for ECMO use in pregnancy or the peripartum period, systematic reviews or analysis of large administrative data sets have reported survival rates ranging from 69.5% to 79.3%.^5,6,9,22^ In the current investigation of COVID-19 ARDS, the maternal survival rate was higher at 84%. This survival rate is greater than the 61% that has been reported for in-hospital patients receiving ECMO.^23^ Our findings are similar to other recent studies noting survival rates in pregnancy for patients receiving ECMO due to COVID-19 infection of 84% to 86.7%.^8,24^

Previous studies of thromboembolic events in pregnant patients on ECMO before the COVID-19 pandemic have reported ranges between 2.8% to 33.0%.^6,9^ However, a more recent report of pregnant patients receiving ECMO with COVID-19 infection noted a rate of 40%, similar to the current analysis.^24^ In the current study, we cannot determine the degree of anticoagulant therapy when the thromboembolic event occurred in patients receiving ECMO therapy. The thromboembolic effects of the SARS-CoV-2 virus have been documented throughout the pandemic,^4^ and may have played a role in the unexpectedly high rate in the present cohort.

One must interpret cautiously the association of increased mortality in patients cared for at institutions with less than 3 reported ECMO cases in pregnancy with severe COVID-19 infection. Overall center ECMO volumes were not reported and we cannot determine the acuity of when the pregnant individual presented, if comorbidities existed that exasperated respiratory failure, or when a patient may have been transferred to a higher level of care institution.

Finally, not surprisingly, one of the comorbidities associated with an increased risk of maternal mortality was cardiac arrest in a pregnant individual receiving ECMO. Similar increased mortality associated with cardiac arrest has been noted in pregnant patients receiving ECMO for other indications unrelated to COVID-19 infection. In an analysis of the National Inpatient Sample from 2010 to 2016, pregnant patients receiving ECMO who experienced cardiogenic shock or circulatory arrest had a mortality rate of 42%.^10^

The study’s strengths are that it is a large, multicenter trial that spans multiple hospital systems—academic, community, and military. The study population is heterogenous and therefore generalizable to the US population and contemporary ECMO management. Additionally, systematic direct data extraction from the medical record was performed, which allowed for the collection of granular data on the maternal-fetal dyad and a detailed assessment of morbidity events that could not be evaluated using administrative or billing data.

### Limitations

We note several limitations of the study. First, multiple investigators entered data into the REDCap registry, which may increase the possibility of incorrect or incomplete data. The data were checked for accuracy and investigators were queried for any missing variables. Second, the study’s retrospective nature and the limitation of electronic medical record documentation dictated what data could be collected. Third, a correction for multiple comparisons was not performed because this was an exploratory analysis for hypothesis generation. Fourth, given the rare event of ECMO initiation during the peripartum period, some of the absolute risks of adverse maternal and neonatal outcomes are low, so the possibility of a type II error may exist. Additionally, maternal fatality data may be incomplete, as death may have occurred 30 days after discharge or at a different facility. Furthermore, this study did not have a control group of nonpregnant patients available for comparison; therefore, additional studies may be needed to better understand the effect that ECMO may have on the pregnant or postpartum patient.

## Conclusions

In this multicenter US cohort study, most pregnant and postpartum individuals who required ECMO for COVID-19–associated respiratory failure survived, but experienced high frequency of serious maternal morbidity and pregnancy loss events. These findings suggest that pregnant and postpartum patients should be considered good candidates for ECMO initiation for COVID-19–associated respiratory failure.

## Notes

Supplement 1. **eTable 1.** Obstetrical and Perinatal Outcomes by Center Volume **eTable 2.** Potential Factors Associated With Maternal Death

Supplement 2.
Data Sharing Statement
