# Error in Results

**DOI:** 10.1001/jamanetworkopen.2024.15971

**Published:** 2024-05-09

In the Original Investigation titled “Outcomes Following Extracorporeal Membrane Oxygenation for Severe COVID-19 in Pregnancy or Post Partum,”^1^ published May 22, 2023, there was a sentence in the Results section that should have read, “Among the 23 patients (23.0%) who delivered while receiving ECMO, the median (IQR) gestational age was 29.0 (26.9-29.6) weeks, and 8 (34.8%) had a postpartum hemorrhage.” This article has been corrected.^1^
