# Supplementary material for: Outcomes Following Extracorporeal Membrane Oxygenation for Severe COVID-19 in Pregnancy or Post Partum
Source: JAMA Netw Open. 2023 May 22;6(5):e2314678. doi: 10.1001/jamanetworkopen.2023.14678 (PMC10203887; doi:10.1001/jamanetworkopen.2023.14678)
Supplement: Supplement 1. — eTable 1. Obstetrical and Perinatal Outcomes by Center Volume eTable 2. Potential Factors Associated With Maternal Death [file jamanetwopen-e2314678-s001.pdf]

Supplemental Online Content

Byrne JJ, Shamshirsaz AA, Cahill AG, et al. Outcomes following extracorporeal membrane oxygenation for severe COVID-19 in pregnancy or post partum. *JAMA Netw Open.* 2023;6(5):e2314678. doi:10.1001/jamanetworkopen.2023.14678

eTable 1. Obstetrical and Perinatal Outcomes by Center Volume

eTable 2. Potential Factors Associated With Maternal Death

This supplemental material has been provided by the authors to give readers additional information about their work.

© 2023 Byrne JJ et al. *JAMA Network Open.*

eTable 1. Obstetrical and Perinatal Outcomes by Center Volume

Variable	≤ 3 (n=14)	≥ 4 (n=84)	P-value[a]
Maternal death	5 (35.7)	11 (13.1)	0.05
Intracranial hemorrhage	1 (7.1)	4 (4.8)	0.55
Hemolysis	2 (14.3)	8 (9.5)	0.63
Coagulopathy	4 (28.6)	14 (16.7)	0.28
HIT	0 (0.0)	4 (4.8)	1.00
Need for CRRT	2 (14.3)	7 (8.3)	0.61
Liver failure	1 (7.1)	0 (0.0)	0.14
Gastrointestinal bleeding	2 (14.3)	5 (6.0)	0.26
Limb ischemia	1 (7.1)	1 (1.2)	0.27
Venous thromboembolism	2 (14.3)	35 (41.7)	0.07
Stroke	1 (7.1)	4 (4.8)	0.55
Ischemic injury (non-cardiac or intracranial)	2 (14.3)	6 (7.1)	0.32
Acute kidney injury	5 (35.7)	21 (25.0)	0.51
Myocardial Infarction	2 (14.3)	1 (1.2)	0.05
Cardiomyopathy	1 (7.1)	8 (9.5)	1.00
Arrhythmias	1 (7.1)	16 (19.0)	0.45
Cardiogenic Shock	1 (7.1)	3 (3.6)	0.47
Cardiac arrest	3 (21.4)	12 (14.3)	0.45
Hypertensive disorders of pregnancy	5 (35.7)	27 (32.1)	0.77
Placental abruption	0 (0.0)	1 (1.2)	1.00
Preterm prelabor rupture of membranes	0 (0.0)	1 (1.2)	1.00
Early pregnancy loss	2 (14.3)	3 (3.6)	0.15
Fetal demise	2 (14.3)	5 (6.0)	0.27
Neonatal demise	0 (0.0)	3 (3.6)	1.00
Small for gestational age	0 (0.0)	6 (7.2)	0.59
Birth weight <1500 grams	5 (50.0)	29 (51.8)	1.00
Birth weight <2500 grams	6 (60.0)	42 (75.0)	0.44
Apgar <3 at 5 min	2 (14.3)	10 (12.2)	0.69
Neonatal ICU admission	6 (66.7)	48 (75.0)	0.69
Neonatal intubation	3 (42.9)	37 (63.8)	0.41
Respiratory distress syndrome	4 (66.7)	35 (60.3)	1.00
Transient tachypnea of the newborn	0 (0.0)	4 (6.9)	1.00
Neonatal sepsis	0 (0.0)	3 (5.2)	1.00
Interventricular hemorrhage	1 (16.7)	4 (6.9)	0.40
Necrotizing enterocolitis	0 (0.0)	3 (5.2)	1.00
Bowel perforation	0 (0.0)	1 (1.7)	1.00
Bronchopulmonary dysplasia	1 (16.7)	9 (15.5)	1.00
Chronic lung disease	3 (50.0)	3 (5.2)	**0.01**

[a]Fisher's Exact Test

© 2023 Byrne JJ et al. *JAMA Network Open.*

eTable 2. Potential Factors Associated With Maternal Death

Variable	Alive (n=82)	Dead (n=16)	P-value[a]
Age	32.0 [27.3, 34.0]	34.5 [28.5, 37.3]	0.10[b]
BMI	35.7 [30.4, 40.2]	36.4 [33.58, 39.5]	0.71[b]
Parity	2.0 [1.0, 3.0]	2.0 [1.0, 3.0]	0.52[b]
Social Vulnerabilty score	3.0 [2.0, 4.0]	3.0 [1.8, 4.0]	0.78[b]
Intracranial hemorrhage	2 (2.4)	3 (18.8)	0.03
Hemolysis	8 (9.8)	2 (12.5)	0.67
Coagulopathy	14 (17.1)	4 (25.0)	0.49
HIT	3 (3.7)	1 (6.2)	0.52
Need for CRRT	7 (8.5)	2 (12.5)	0.64
Liver failure	0 (0.0)	1 (6.2)	0.16
Gastrointestinal bleeding	5 (6.1)	2 (12.5)	0.32
Limb ischemia	2 (2.4)	0 (0.0)	1.00
Venous thromboembolism	34 (41.5)	3 (18.8)	0.10
Stroke	3 (3.7)	2 (12.5)	0.19
Ischemic injury (non-cardiac or intracranial)	6 (7.3)	2 (12.5)	0.61
Acute kidney injury	17 (20.7)	9 (56.2)	**0.01**
Cardiac event	34 (41.5)	10 (62.5)	0.17
Myocardial Infarction	2 (2.4)	1 (6.2)	0.42
Cardiomyopathy	8 (9.8)	1 (6.2)	1.00
Arrhythmias	16 (19.5)	1 (6.2)	0.29
Cardiogenic Shock	4 (4.9)	0 (0.0)	1.00
Cardiac arrest	7 (8.5)	8 (50.0)	**<0.01**
Other Cardiac	8 (9.8)	2 (12.5)	0.67
Hypertensive disorders of pregnancy	29 (35.4)	3 (18.8)	0.25
Placental abruption	1 (1.2)	0 (0.0)	1.00
Preterm prelabor rupture of membranes	1 (1.2)	0 (0.0)	1.00
Other Complications	30 (36.6)	6 (37.5)	1.00
Early pregnancy loss	2 (2.4)	3 (18.8)	**0.03**
Fetal demise	7 (8.6)	0 (0.0)	0.60
Neonatal demise	2 (2.5)	1 (6.2)	0.42

[a]Fisher's Exact Test
[b]Mann Whitney U Test

© 2023 Byrne JJ et al. *JAMA Network Open.*
