# Supplementary material for: Outcomes Following Extracorporeal Membrane Oxygenation for Severe COVID-19 in Pregnancy or Post Partum
Source: JAMA Netw Open. 2023 May 22;6(5):e2314678. doi: 10.1001/jamanetworkopen.2023.14678 (PMC10203887; doi:10.1001/jamanetworkopen.2023.14678)
Supplement: Supplement 2. — Data Sharing Statement [file jamanetwopen-e2314678-s002.pdf]

Data Sharing Statement

Byrne. Outcomes Following Extracorporeal Membrane Oxygenation for Severe COVID-19 in Pregnancy or Post Partum. *JAMA Netw Open*. Published May 22, 2023. doi:10.1001/jamanetworkopen.2023.14678

Data
Data available: No
